# Susceptibility of mosquito vectors of the city of Praia, Cabo Verde, to Temephos and *Bacillus thuringiensis* var *israelensis*

**DOI:** 10.1371/journal.pone.0234242

**Published:** 2020-06-10

**Authors:** Sílvia Pires, Joana Alves, Ibrahima Dia, Lara F. Gómez

**Affiliations:** 1 Unidade de Ciências da Natureza, da Vida e do Ambiente, Universidade Jean Piaget de Cabo Verde, Praia, Cabo Verde; 2 Instituto Nacional de Saúde Pública, Ministério da Saúde, Praia, Cabo Verde; 3 Unité d’entomologie médicale, Institut Pasteur de Dakar, Dakar, Senegal; National Taiwan Ocean University, TAIWAN

## Abstract

Many vector-borne diseases circulate in the Republic of Cabo Verde. These include malaria during the colonization of the archipelago by the Portuguese explorers and several arboviruses such as yellow fever (now eradicated), dengue and zika.

To control these vector-borne diseases, an integrated vector control program was implemented. The main targeted mosquito vectors are *Aedes aegypti* and *Anopheles arabiensis*, and in a lesser extent the potential arbovirus vector *Culex pipiens* s.l. The main control strategy is focused on mosquito aquatic stages using diesel oil and Temephos. This latter has been applied in Cabo Verde since 1979. Its continuous use was followed by the emergence of resistance in mosquito populations.

We investigated the current susceptibility to Temephos of the three potential mosquito vectors of Cabo Verde through bioassays tests. Our results showed various degrees of susceptibility with 24h post-exposure mortality rates ranging from 43.1% to 90.9% using WHO diagnostic doses. A full susceptibility was however observed with *Bacillus thurigiensis* var *israelensis* with mortality rates from 99.6% to 100%.

## Introduction

Mosquitoes (*Diptera*: *Culicidae*) are insects of the greatest importance to global health, because, in addition to the discomfort caused by their bites, they transmit a wide variety of pathogens [1] and represent therefore a major public health problem. Overall, 17% of the recorded diseases worldwide are caused by mosquitoes with 700,000 deaths per year [2].

In Cabo Verde, several mosquito-borne diseases have been recorded: Yellow fever, lymphatic filariasis, malaria, dengue fever and zika [3–8]. Some of them were endemic in the country for a long time. It is particularly the case of malaria which was identified since the 15th century at the time of the settlement of the archipelago. It is transmitted by *Anopheles arabiensis*, a member of the *Anopheles gambiae* complex, which was the first mosquito described in Cabo Verde in 1909 [9]. Despite the decrease of the number of malaria cases during the last decades, malaria is not yet completely eradicated in the country. Even if it was almost eradicated between 1954 and 1970, it still represents a main public health problem. Currently, the country is considered by the WHO to be in the pre-eradication phase [10], with a short-term goal of eliminating this disease by 2020 [11]. In recent previous years, the disease was limited to the Santiago Island with more than 400 indigenous cases and one death recorded during the last epidemic in 2017 [12–15].

For the other mosquito-borne diseases, Cabo Verde was exposed to various arboviruses. In 2009, the archipelago experienced the emergence of dengue virus serotype III. This arbovirus caused the largest epidemic in West Africa with more than 21,000 cases [6, 7]. *Ae*. *aegypti* was identified as the main vector during this epidemic. Its presence on the archipelago was reported since 1945 [16]. In 2015, it was incriminated as the main vector during an epidemic of zika with about 8000 cases [8].

Due to the absence of vaccines or specific treatments for these diseases, vector control is an effective and valuable alternative to control these diseases. It is based on the use of various methodologies and/or tools [17–21]. Among these tools, those based on the use of chemicals as insecticides are most used to control mosquito populations both at larval and adult stages. In Cabo Verde, Temephos is the most widely used for mosquito control with other techniques including diesel oil, predatory fishes in mosquito breeding sites and indoor residual spraying with deltamethrin [13].

As observed elsewhere, with the continuous use of chemical compounds for vector control, the main limitation is the emergence of resistant mosquito populations [22–25]. For the specific case of Temephos, its use since 1979 [26, 27] was followed by the apparition of resistant *Ae*. *aegypti* populations on the island of Santiago in 2012 and 2014 [28]. Similarly, pyrethroid resistance was also observed in *Anopheles* with the detection of the resistant alleles from molecular studies. For Temephos, the mechanism involved in the resistant populations are not yet identified and need further studies as well as in *Culex* vectors [29].

To face this resistance, *Bacillus thuringiensis* var *israelensis* (Bti), a biolarvicide, has been proposed as an alternative to overcome the observed resistance [30, 31]. Its low residual effect on the environment [32] and its effectiveness has been demonstrated in several countries including Cabo Verde [28]. Therefore, we evaluated in this study its effectiveness against three mosquito vectors compared to doses of Temephos used by health agents in Cabo Verde and recommended by WHO. The final goal was to use it as an alternative to overcome the resistance with Temephos.

## Materials and methods

### Study area and sampling sites

**Fig 1:**
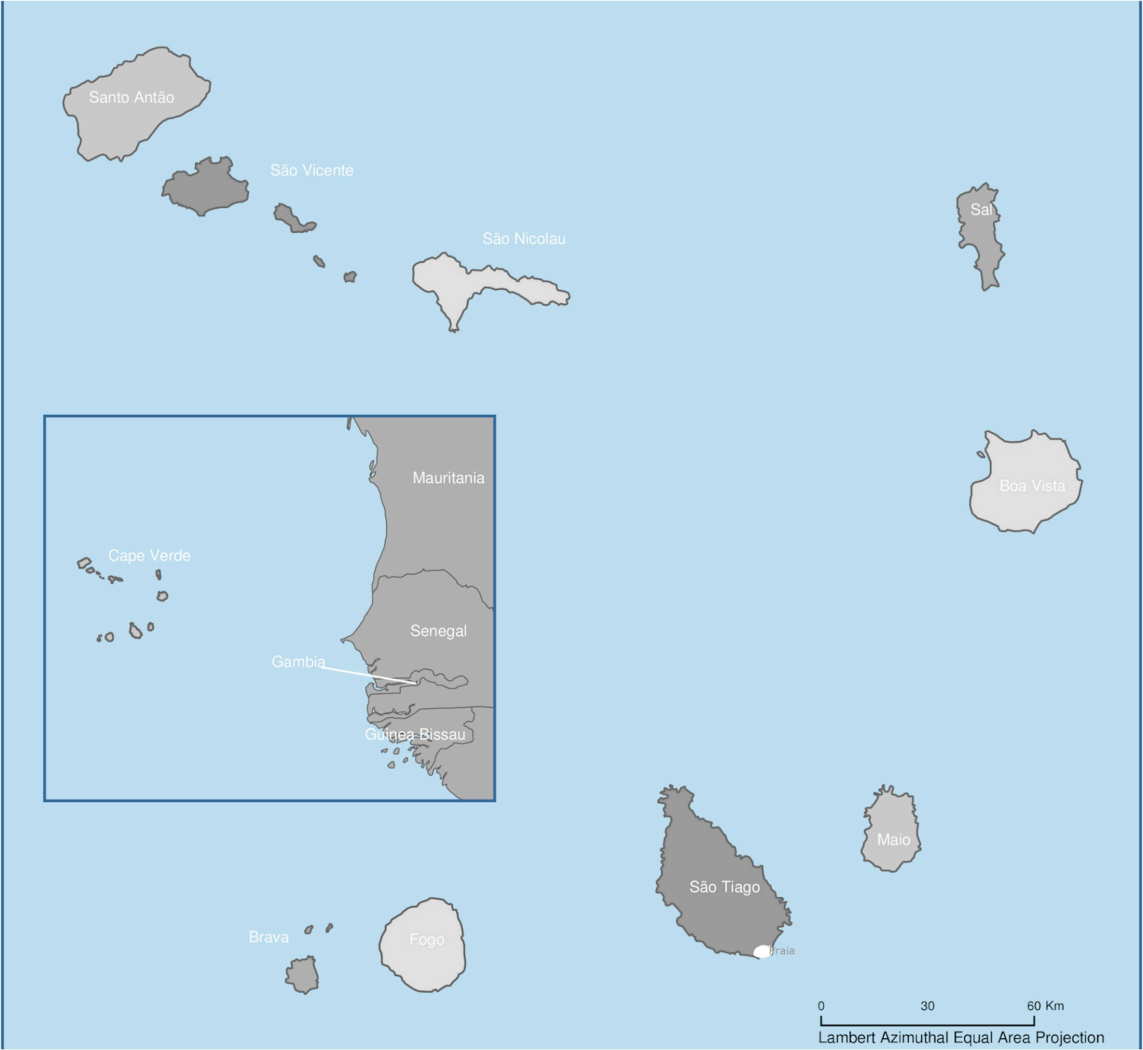
Cabo Verde, Santiago Island, city of Praia. This map was adapted from an image extracted from https://sedac.ciesin.columbia.edu/data/collection/gpw-v3/maps/gallery/search?contains=Cape+Verde for illustrative purposes only. City of Praia is marked in white in the south of Santiago Island.

This study was carried out in the city of Praia, the main urban area of the island of Santiago, located on the western coast of the African continent in the Atlantic Ocean, between latitude 14° and 18° N and longitude 22° and 26° W (Fig 1).

The climate of the region is subtropical dry with an arid season during most part of the year and a short rainy season that lasts from July to October. The average annual rainfall estimate is between 300 and 700 mm. The average mean annual temperature is 25° C [33].

**Fig 2:**
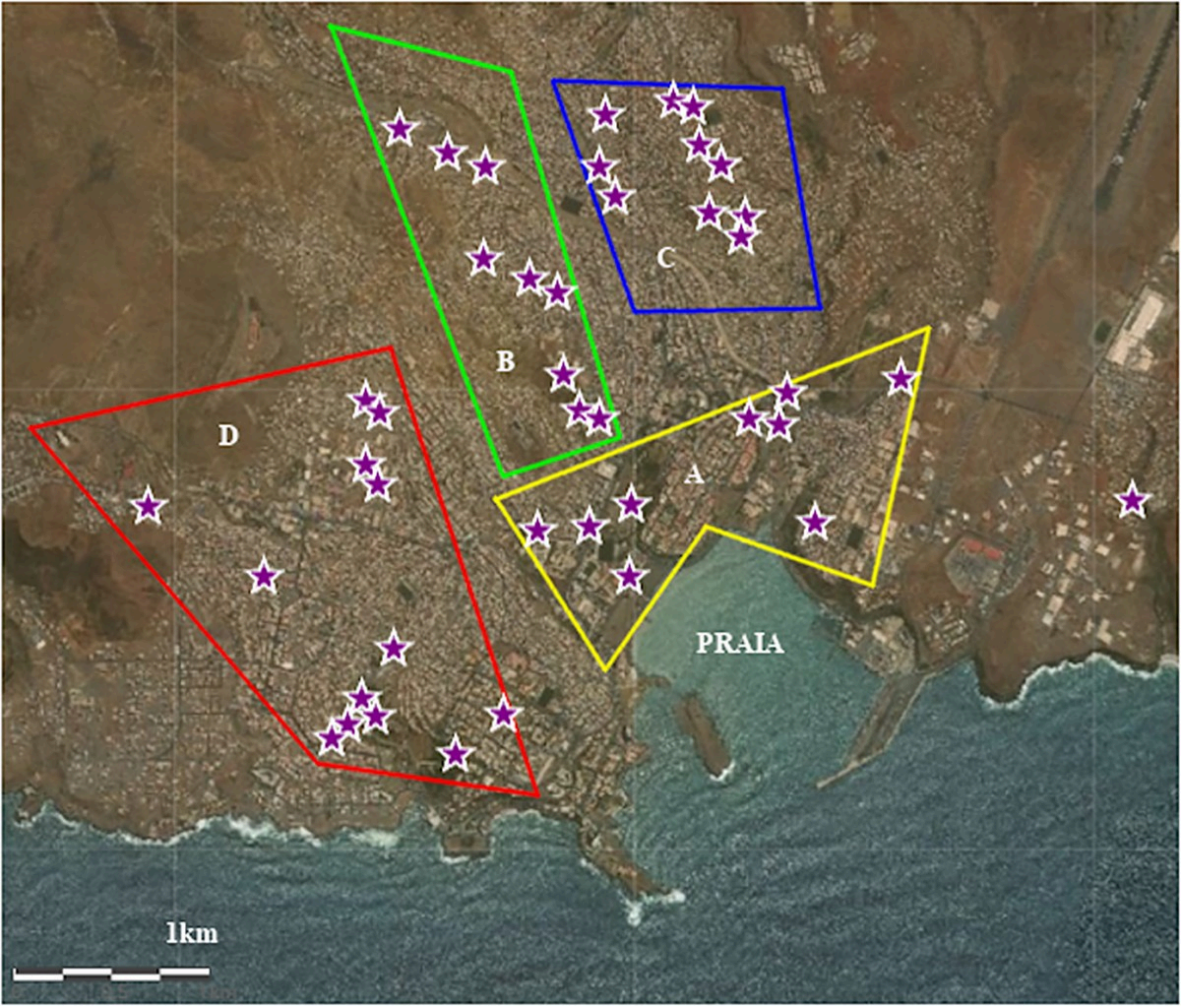
Sampling sites of *Anopheles* spp., *Cx*. *pipiens* s.l. and *Ae*. *aegypti* in the city of Praia. This map was adapted from an extracted from image http://idecv-ingt.opendata.arcgis.com/datasets/cartografia-st-2010 for illustrative purposes only. Sampling sites. The polygons marked A (yellow), B (green), C (blue) and D (red) represent the four collection zones, of culicids, in the City of Praia. The sites are marked with violet coloured stars.

*Ae*. *aegypti*, *Cx*. *pipiens* s.l. and *Anopheles* spp. eggs and larvae were collected respectively using BR-OVT ovitraps and by larval collection in the city of Praia (Fig 2).

The area was divided into 4 zones (A, B, C and D), each zone containing several sites in the city of Praia (see Table 1).

**Table 1:** Distribution of the sites of the city of Praia through the sampling zones.

Zones	Sites investigated
Zone A	Achada Grande Frente, Lém-Ferreira, Gamboa, Várzea.
Zone B	Eugénio Lima, Achadinha Pires, Bairro
Zone C	Ponta d´Água, Vila Nova, Pensamento
Zone D	Achada Santo António, Tira-chapéu, Terra Branca, Palmarejo

The sites were selected in different places including public spaces of the city and in small agricultural lands. Apart from these latter sites for which permission was obtained from owners, no permission was necessary for the collections in public spaces. Field studies did not involve endangered or protected species.

In each site, the collection points were chosen within the zones based on the following criteria: (1) For *Cx*. *pipiens* s.l. and *Ae*. *aegypti*, we privileged the presence of vegetation and/or agricultural fields, with pools of stagnant water and high density of people such as the presence of schools and health centres. (2) For *Anopheles* spp., the samples were collected mainly in the locations of Achada Grande Trás and Várzea, characterized by a larger number of small temporary freshwater pools during the rainy season, where mainly breed Anopheline larvae.

### Samples collection and treatment

#### Egg and larval collections

Ae. aegypti and Cx. pipiens s.l. larvae were obtained from hatched eggs collected by the oviposition traps BR-OVT [34]. These traps were supplied with acacia infusion as attractant [35] and were installed in the different places selected in the city of Praia. They were inspected and once eggs were collected, they were taken to the laboratory and the eggs placed in white plastic containers trays containing water for hatching. Upon hatching, respectively between 30 and 60 larvae or 125 to 250 larvae were kept and reared with 200 ml or 500 ml of chlorinated tap water.

**Fig 3:**
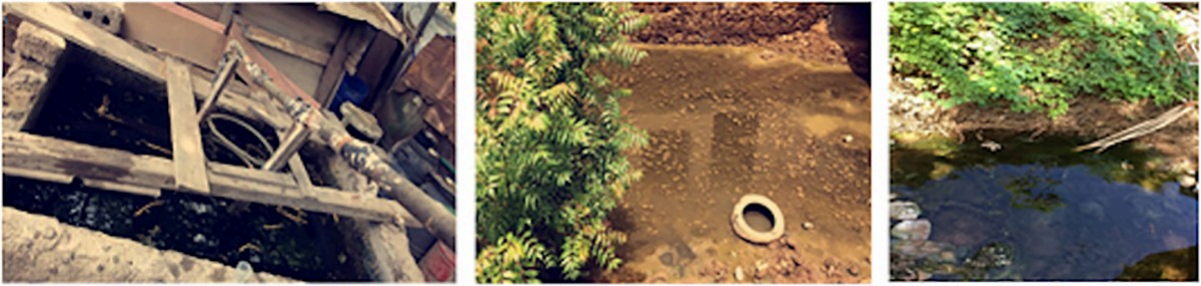
*Anopheles* spp. and *Culex pipiens* s.l. breeding sites. **A.** *Culex pipiens* s.l. artificial breeding site. **B**. *Anopheles* spp. natural breeding site. **C**. *Anopheles* spp. artificial breeding site.

*Anopheles* spp. and *Cx*. *pipiens* s.l. larvae were collected in natural or artificial breeding sites using nets for larvae or buckets with or without a light source (Fig 3).

#### Maintenance and identification of larvae

The larvae were kept in laboratory and were fed with crushed and autoclaved flocculated fish food. The amount of daily food for larvae of the L1 and L2 stages was 0.003g and for the L3 and L4 stages 0.006g per plastic container. The water was removed and replaced every 3 days. Larvae were maintained in standard conditions at a temperature of 25±2°C, 75±10% relative humidity and 12:12h photoperiod [36].

They were identified using the taxonomic key of mosquitoes in Cabo Verde [16].

### Larvicides

The biological larvicide tested in the bioassays was *Bacillus thuringiensis* var *israelensis*, strain AM65-52, 37.4% (w/w) as dispersible granule, manufactured by |Valent BioSciences Corporation, batch nº| 246-846-PG. The doses used were 3, 7, 11 and 15 kg/ha, corresponding respectively to the minimum dose, mean dose 1, mean dose 2 and maximum dose recommended by the manufacturer and adjusted to the surface of the container.

The chemical larvicide tested in this study was Temephos 1% granulated, manufactured by SDS Ramcides Hop Science Pvt. Ltd, lot #: SDSREP111601. The concentrations used were of 0.25 mg/L as recommended by WHO and the manufacturer dose) and 1 mg/L as used and applied by health agents in routine larval control activities in Cabo Verde) and adjusted to the volume of the container.

### Bioassays tests for *Ae*. *aegypti* and *Cx*. *pipiens* s.l.

For each larvicide, the assays were performed using 40 L3/L4 stage larvae in three replicates. Each larvicide was dissolved directly into 200 ml of dechlorinated water. Fifteen minutes after dissolution, the physiochemical parameters of the water (pH, temperature, salinity, conductivity and dissolved organic materials) were measured using PCSTestr TM 35 portable multiparameter.

All the experiments were carried out in standard conditions at a temperature of 25±2°C, 75±10% relative humidity and 12:12h photoperiod.

The full protocol is available at dx.doi.org/10.17504/protocols.io.bbstinen

### Bioassays tests for *Anopheles* spp.

To analyse the susceptibility of *Anopheles* spp. for each larvicide, bioassays were performed with the same methodology explained for *Ae*. *aegypti* and *Cx*. *pipiens* s.l.

Using the WHO method [37], the bioassays were repeated only for Temephos at the concentration of 1 mg/L, which corresponds to the dose applied by health agents in Cabo Verde. The bioassays were performed for 25 larvae from L3/L4 stage in 100 ml, in four replicates without food. After 24 hours of exposure, the number of dead and dying larvae in each replicate was recorded. The bioassay was repeated three times at different dates between September and October 2017.

For each assay, larvae collected from the different sites were used as control.

The quality control of Temephos used in this study was evaluated using a susceptible strain, *Anopheles coluzzii*, maintained at the Medical Entomology Unit of Dakar Pasteur Institute.

Using the WHO method [37], bioassays were performed to analyse the susceptibility of *Anopheles* spp. to 1 mg/L of Temephos, taking into account different physical and chemical factors namely feeding, type of water or the area of collection of the larvae.

For the evaluation of the feeding effect of the larvae on the bioassay test, the larvae were pooled using as control larvae for which no food was supplied.

The effect of the type of water on the bioassay was studied using three types of water: dechlorinated tap water, mineral water and water from natural breeding site. Dechlorinated tap water was used as control because it was used along all the bioassays.

For the evaluation of the effect of the origin of *Anopheles* spp., larvae of L3/L4 stages were collected from Achada Grande Trás and Várzea.

### Statistical analysis of data

Sampling data were collected in field records: BROVT form and larval inspection form. The data were entered into a Microsoft Office Excel 2016 database and the Ovitrap Positivity Index (POI) and the Egg Density Index (IDO) were calculated according to [38].

The effectiveness of the larvicides were assessed 24 hours post-exposure using the total number of dead larvae. The assay was considered invalid when the mortality of in the control was >10%. When mortality was between 5 and 10%, the ABBOTT mortality correction formula was applied [39]. The test was discarded when more than 10% of pupae was obtained or the mortality in the negative control was 20% or more.

All the data were recorded in field sheets and stored into a Microsoft Office Excel 2016 database. The mortality rate was calculated as the percentage of dead larvae from all replicates for Temephos and Bti. For the different mean, standard deviation and standard error were calculated.

To analyse the robustness of the results obtained and due to the existence of a high number of zeros, a Zero-inflated Poisson regression (ZIP) model and Zero-inflated Negative Binomial regression (ZINB) were used. Respectively, the number of surviving larvae in the bioassays was used as dependent variable and the insecticide tested as exposure variable. Bivariate and multivariate adjustments were made with the independent variables; number of replicates per bioassay and number of bioassays performed for each genus/species of mosquito. For all tests a p value <0.05 was considered as statistically significant. The application of the ZIP model was considered when the values obtained for the probability of X2 were greater than 0.005 (Prob> X2). The ZIP-likehood ratio test was used to evaluate the application of the ZINB model. A significant likelihood ratio test for the overdispersion parameter, *alpha* = 0 indicates that the ZINB model is preferred to the ZIP model. The software Stata V.14.0 was used for the statistical analyses.

## Results

### Sample collections

A total of 4633 *Ae*. *aegypti* were collected through by the BR-OVT ovitraps among 36 of which 33 were positive (presence of eggs), giving an ovitrap positive index (POI) of 91% (Table 2). For *Cx*. *pipiens* s.l., 48 rafts were collected (15 in BR-OVT and 33 directly from breeding sites). For *Anopheles* spp. all larvae were collected by direct larval search.

**Table 2:** Number of ovitraps, total number of eggs, ovitraps positivity index (POI) and eggs density index (EDI) of *Ae*. *aegypti*.

	Zone A	Zone B	Zone C	Zone D	Zones A-D
**BR-OVT**	7	10	10	9	**36**
**Positive BR-OVT**	6	9	10	8	**33**
**Number of Eggs**	1039	1052	1646	896	**4633**
**POI (%)**	86	90	100	89	**91**
**EDI**	173	117	165	112	**142**

### Bioassays of susceptibility to larvicides

The Temephos and Bti larvicides were tested in *Ae*. *aegypti* mosquito populations, *C*.*x pipiens* s.l. and *Anopheles* spp. from the City of Praia, Cabo Verde, in two different periods. The mean mortality rates for each concentration of each insecticide were analysed separately for each mosquito species.

For larvae bioassays, *Ae*. *aegypti* larvae were selected from eggs collected from the 4 collection sites in Praia (Fig 2). For *Cx*. *pipiens* s.l. larvae were selected from the egg rags hatching collected mainly from zone D, directly from the breeding sites (Figs 2 and 3). For *Anopheles* spp., larvae were selected from the collections made directly from breeding sites located in Várzea and Achada Grande Trás (Fig 3).

**Fig 4:**
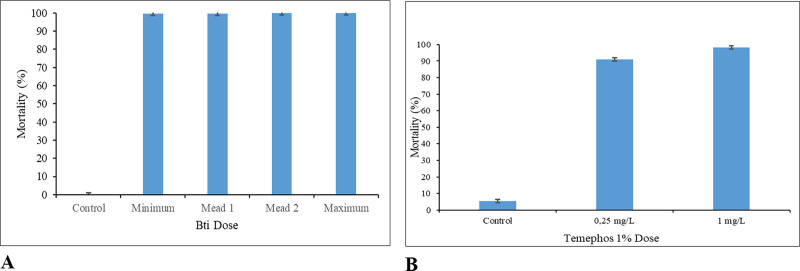
Mean mortality of *Ae*. *aegypti* by chemical and biological insecticides. **A.** Mean mortality of *Ae*. *aegypti* by Bti. Minimum dose– 3 Kg/ha, mean dose 1–7 Kg/ha, mean dose 2 – 11Kg/ha and maximum dose– 15Kg/ha. **B.** Mean mortality of *Ae*. *aegypti* by Temephos. For each set of data, the standard error (black colour) is displayed.

For *Ae*. *aegypti*, the mortality rates for Bti were from 99.6% to 100% after 24 hours of exposure. For Temephos, the reported mortality rates were 90.9% at the dose recommended by the WHO and 98.2% at dose used by the health agents in Cabo Verde, after 24h (Fig 4A and 4B).

**Fig 5:**
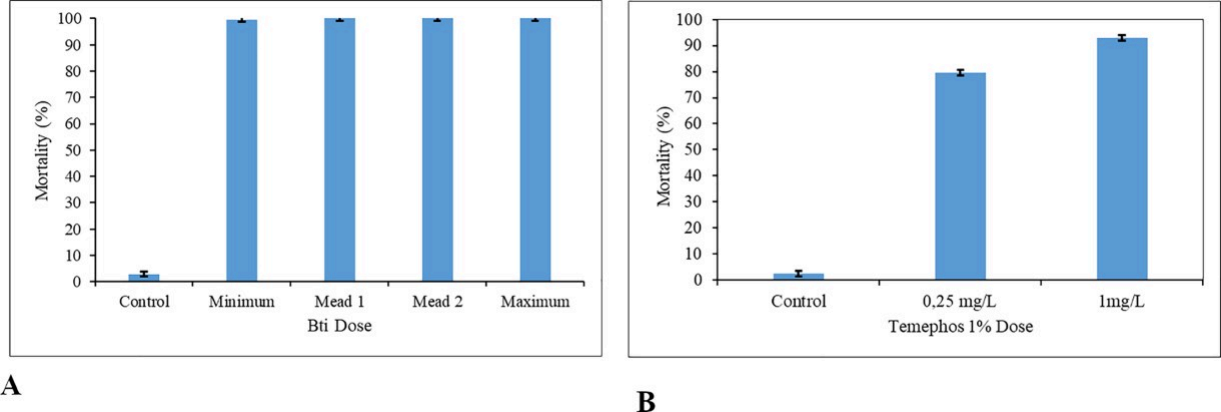
Mean mortality of *Culex pipiens* s.l. by chemical and biological insecticides. **A.** Mean mortality of *Culex pipiens* s.l. by Bti. Minimum dose– 3 Kg/ha, mean dose 1–7 Kg/ha, mean dose 2–11 Kg/ha and maximum dose– 15 Kg/ha. **B.** Mean mortality of *Culex pipiens* s.l. by Temephos. For each set of data, the standard error (black colour) is displayed.

For *Cx*. *pipiens* s.l. the mortality rates for Bti ranged from 99.6% to 100%. For Temephos the respective mortality rates at the dose recommended by WHO and that used by the health agents were 79% and 92.9% (Fig 5A and 5B).

**Fig 6:**
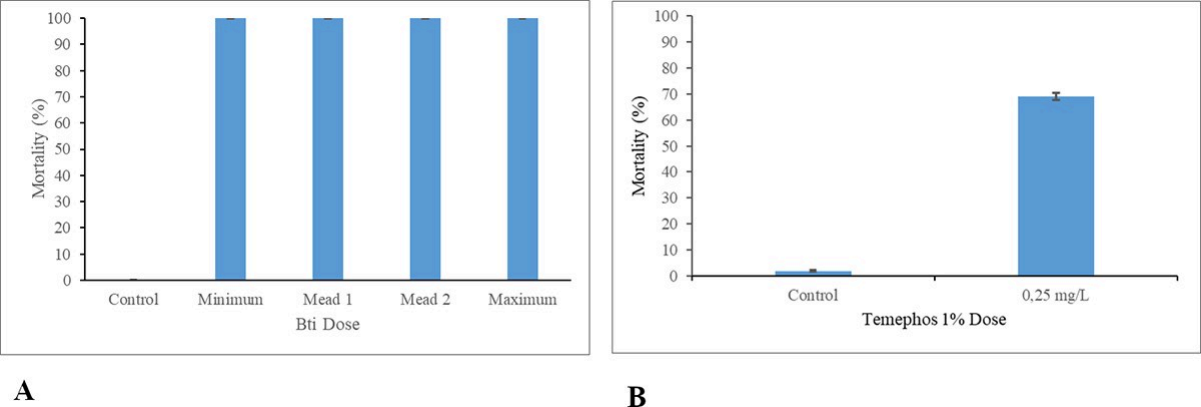
Mean mortality of *Anopheles* spp. by chemical and biological insecticides. **A.** Mean mortality of *Anopheles* spp. by Bti. Minimum dose– 3 Kg/ha, mean dose 1–7 Kg/ha, mean dose 2–11 Kg/ha and maximum dose– 15 Kg/ha. **B.** Mean mortality of *Anopheles* spp. by Temephos at 0,25 m (mean of trials 1 and 3). For each set of data, the standard error (black colour) is displayed.

For *Anopheles* spp. The mortality rates after exposure to Bti was 100% for all tested concentrations. In the first bioassay, Temephos was responsible for the mortality of 43.1% of the populations at the dose recommended by WHO and 79.3% at the dose used in Cabo Verde. In the second bioassay, the percentage of pupae was 13.3%. The test was therefore discarded because this percentage was higher than that recommended by WHO (10%). When the test was run with the dose used by the health agents in Cabo Verde, a mortality rate of 58.9% was observed (Fig 6A and 6B).

For the *An*. *coluzzii* laboratory susceptible strain, the mortality rates were respectively 0% and 100% for the control and tested groups during the 9 replicates (3 for the control and 6 for the tested group).

**Fig 7:**
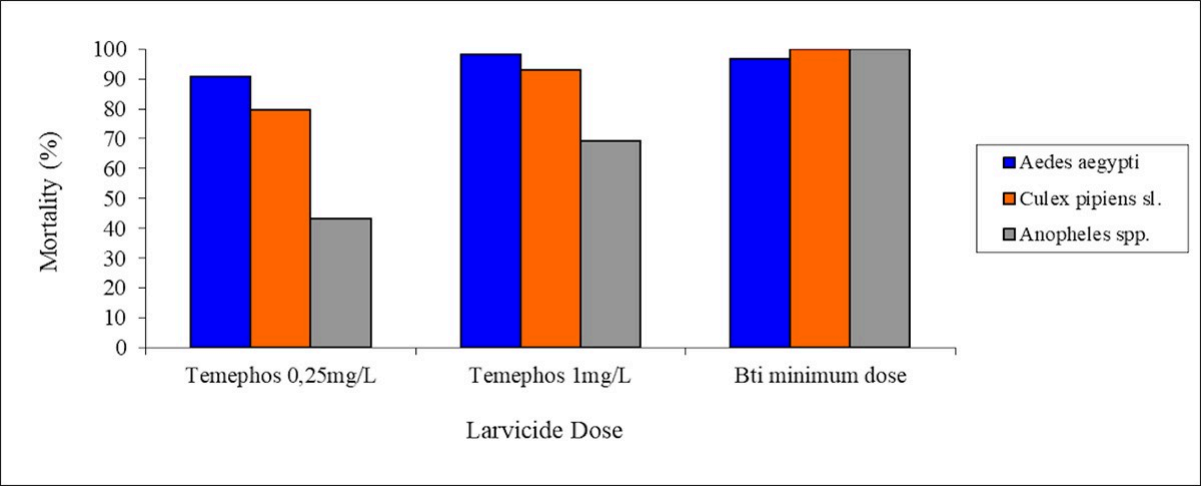
Comparison of mean mortality by Bti, at minimal dose, and by Temephos, among the three species tested. Minimum dose of Bti– 3 Kg/ha. Culicids tested: *Aedes aegypti* in blue colour, *Culex pipiens* s.l. in orange and *Anopheles* spp. in grey colour.

The results of the bioassays demonstrated that the Bti solution, at the minimum concentration recommended by the manufacturer, killed 100% of the L3/L4 larval stages of *Cx*. *pipiens* s.l. and *Anopheles* spp. and 99.6% for *Ae*. *aegypti*. Temephos showed different levels of effectiveness among the species tested (*Ae*. *aegypti*, *Cx*. *pipiens* s.l, *An*. *gambiae* complex and *An*. *pretoriensis*) the bioassays for the two latter species were carried out without separation of the two species of anophelines found in breeding sites. *Ae*. *aegypti* had a higher mean mortality rates than the other for both concentrations with a mortality rate of 90.9% at the dose recommended by WHO. *Cx*. *pipiens* s.l. presented a mortality rate of 79.2% for the dose recommended by WHO. For *Anopheles* spp., the mortality rates were 43.1% at the dose recommended by WHO and 69.1% at the dose applied by health agents in Cabo Verde (Fig 7).

### Evaluation of the susceptibility of *Anopheles* spp. to Temephos

**Fig 8:**
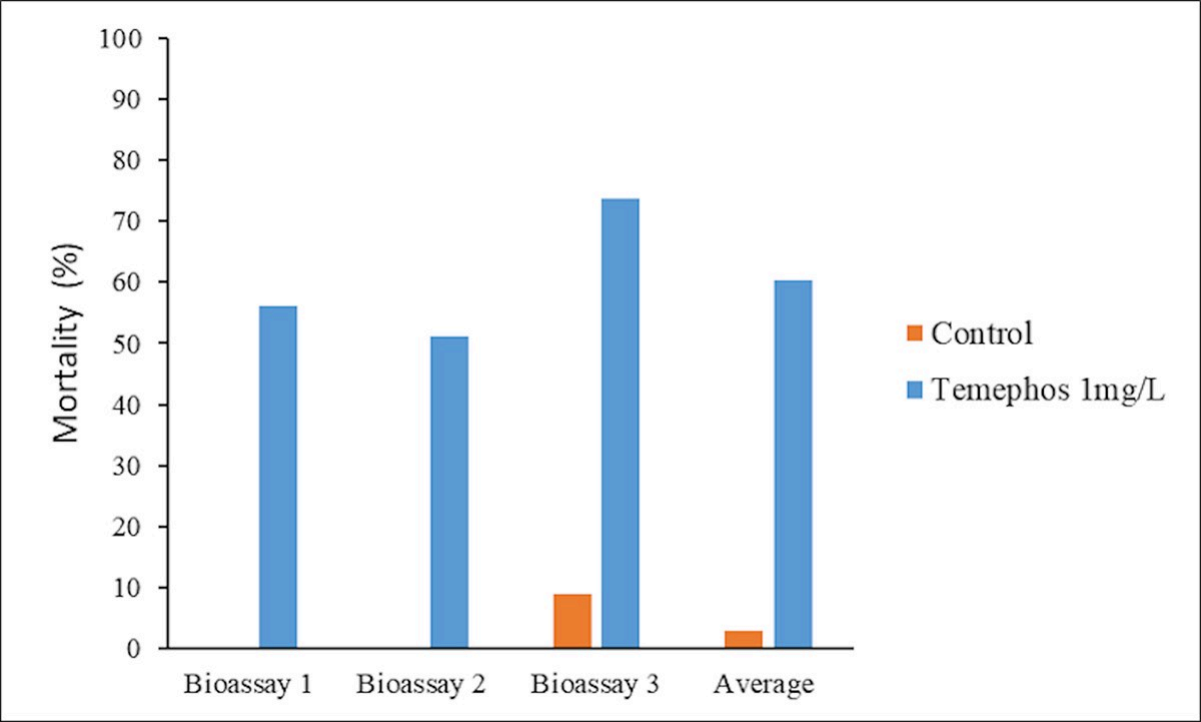
Mortality of *Anopheles* spp. by Temephos, at the rate of application in Cabo Verde, following the WHO method. Temephos rate application in Cabo Verde belong to 1 mg/l. Average columns represent the mean of Bioassay 1, Bioassay 2 and Bioassay 3.

Following the WHO method for performing the Temephos susceptibility bioassays (dose applied in Cabo Verde by the health agents) for *Anopheles* spp., the mortality rates observed were 56%, 51.3% and 73.8% in the bioassays 1, 2 and 3 respectively. These observations showed similar results for the two experimental approaches used, with a minimum mortality of 51.3% and a maximum of 73.8% using the WHO method, whereas the mortality rates observed by the method used and adapted for this study were 58.9% (minimum mortality) and 79.3% (maximum mortality). This allowed to validate the method used in this study (Fig 8).

### Evaluation of the effect of larval feeding, type of water and location of the breeding site, in the susceptibility of *Anopheles* spp. to Temephos

The Table 3 shows the results obtained from the *Anopheles* spp. susceptibility bioassays to Temephos with various larval food and type of water used. The bioassays were done in duplicate. The values presented represent the mean mortalities.

No significant difference was observed for the bioassays for the unfed larvae as well as larvae fed with commercial flocculated autoclaved fish food before the start of the bioassay.

**Table 3:** Effect of feeding and water type used in bioassays on larval mortality of *Anopheles* spp. by Temephos 1%.

Average Mortality (%)					
	Feeding	Without feeding	Dechlorinated Tap Water	Mineral bottled Water	Breeding Water
**Control**	0	0	0	0	0
**Temephos 1mg/L**	51.2	56.0	57.0	65.7	80.2

The mortality rates after exposure to Temephos was compared between the three types of water: mineral water, natural breeding water and dechlorinated tap water (Table 3). The test control was carried out in dechlorinated tap water because this type of water was used in all experiments. The results showed that the difference in *Anopheles* spp. larval mortalities for Temephos were not significant different for the bioassays performed in dechlorinated tap water and mineral water. However, a significant difference was observed for the natural breeding water, with a mortality rate significantly higher than in previous bioassays. A plausible explanation could be the difference observed in the physiochemical parameters of the water of the natural breeding places (Varzea and Achada Grande Trás) in comparison to the other two types (Table 4).

**Table 4:** Physiochemical parameters of water types used in bioassays.

	pH	Temperature (°C)	Salinity (ppm)	TDS (ppm)	Condutivity (μS)
**Várzea breeding water**	7.4	27	615	795	1203
**Achada Grande Trás breeding water**	6–8	28	766	991	1526
**Dechlorinated tap water**	7–3	26	240	334	472
**Mineral bottled water**	7–1	26	24–5	25–4	35–8

To determine if there are some differences in larval mortality of *Anopheles* spp. to Temephos, according to the place of collection of the larvae, we compared the results of the bioassays made with specimens from the two main collection localities namely Várzea and Achada Grande Trás. The respective mortality rates were 34.2% and 59% for Várzea and Achada Grande Trás, with a great difference, which could be attributed to a difference in selective pressure between the two locations by the insecticide Temephos or specific differences between populations.

**Fig 9:**
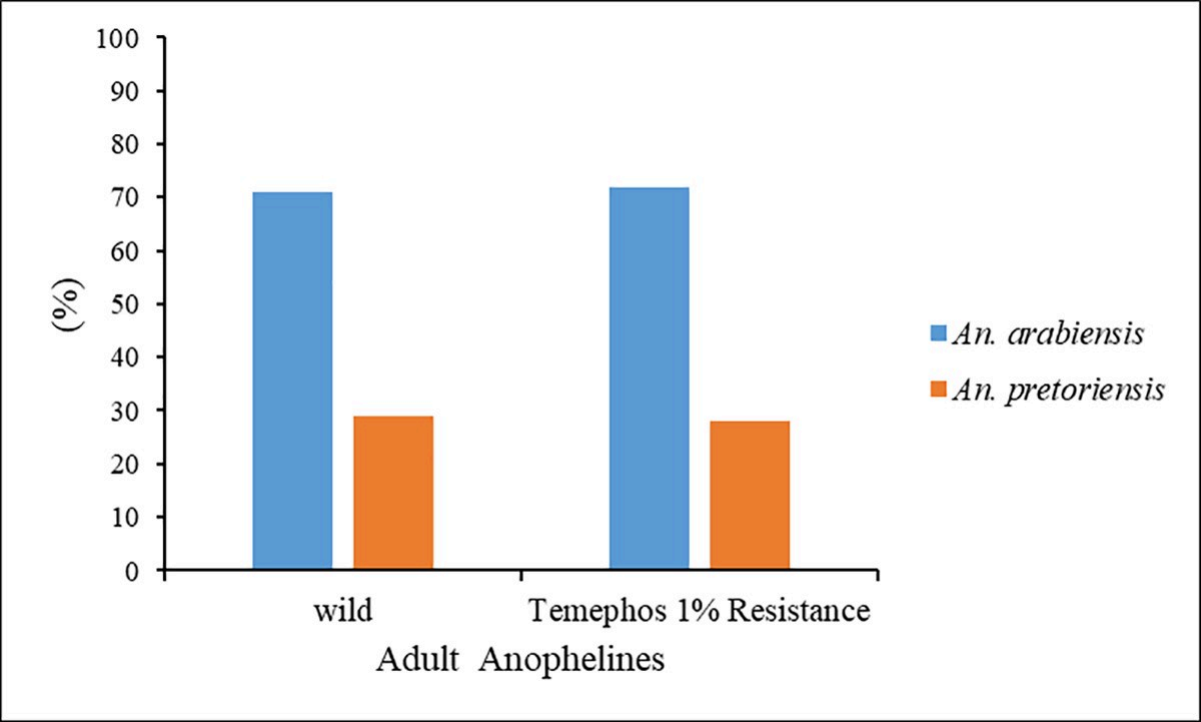
Ratio of *An*. *gambiae* complex and *An*. *pretoriensis* in bioassays with mosquitoes from Achada Grande Trás. Adults anophelines developed from larva control (Wild) and from larvae surviving the Temephos (Temephos 1% Resistance) coming from Achada Grande Trás.

In order to determine which of the two possibilities is more plausible, the surviving mosquitoes from all the experiences with *Anopheles spp*. (control and those exposed to Temephos) from the two localities were kept and reared to adulthood for species identification. In Várzea, 100% of the individuals were identified as *An*. *gambiae* complex from a total of 277 adult mosquitoes (234 wild and 43 Temephos resistant). In the other locality studied, a heterogeneous population was observed with the presence of *An*. *pretoriensis* and *An*. *gambiae* complex (Fig 9).

In Achada Grande Trás, 71% and 29% from 465 susceptible adult mosquitoes and 72% and 28% from 90 Temephos resistant mosquitoes were respectively identified as *An*. *gambiae* complex and *An*. *pretoriensis*. These results confirm the absence of differences in susceptibility to Temephos between the two *Anopheles* species.

### Statistical analysis

The main estimators obtained from the modelling (ZIP and ZINB) are shown in Table 5. The full results of the modelling are presented in the Additional File 2.

**Table 5:** Parameter estimated of the zero-inflated poisson (ZIP) and zero-inflated negative binomial (ZINB) models applied to larvicide susceptibility bioassays.

	Parameters of bivariate ZIP/ZINB models			Parameters of multivariated ZIP/ZINB models		
Variable	^b^Coef (Std Error)	95% ^c^Conf Interval	P value	Coef (Std Error)	95% Conf Interval	P value
**Count Part**						
1. Replica _*Aedes aegypti* Bioassays_	0.062 (0.033)	-0.002–0.126	0.057	0.015 (0.024)	-0.123–0.04	0.283
1. Bioassay _*Aedes aegypti* Bioassays_				0.108 (0.126)	-0.074–0.29	0.245
2. ^a^Replica _*Culex pipiens* s.l. Bioassays_	0.090 (0.048)	-0.004–0,184	0.059	0.199 (0.045)	0.013–0.42	0.013
2. ^a^Bioassay _*Culex pipiens* s.l. Bioassays_				-0.760 (0.260)	0.91- -1.64	0.091
3. Replica _*Anopheles* spp. Bioassays_	^a^0.017 (0.026)	-0.033–0,068	0.500	0.660 (0.033)	0.02–0.13	0.045
3. Bioassay _*Anopheles* spp. Bioassays_				-0.387 (0.239)	-0.885–0.810	0.105
4. ^a^Replica _*Anopheles* spp. WHO Bioassays_	-0.041 (0.02)	-0.080–0,02	0.041	-0.046 (0.020)	-0.085–0.004	0.019
4. ^a^Bioassay _*Anopheles* spp. WHO Bioassays_				0.065 (0.059)	-0.052–0.148	0.269
**Zero Part**						
1. Aedes surviving larvae	-58.38 (0.540)	-59.44- -57.32	0.000	-37.73 (0.697)	-39.10- -36.36	0.000
2. ^a^Culex surviving larvae	-43.02 (23205.52	-45525.01–45438.96	0.999	-42.820 (22003.37)	-43168.64–43083	0.998
3. Anopheles surviving larvae	^a^-39.12 (13102.15)	-25718.86–25640.62	0.998	-40.09 (1.032)	-42.12- -38.07	0.000
4. ^a^Anopheles surviving larvae _WHO Bioassays_	-41.952 (50829.37	-99665.69–99581.59	0.999	-34.12 (7190.89)	-14128.03–14059.78	0.996

Numbers 1, 2, 3 and 4 in the “Variable” column indicate each of the four bioassays in the study.

^a^Results of the Negative binomial Zero-inflated regression Model.

^b^Coef (Std Error) means “Coefficient (Standard error)”

^c^Coef Interval means “Confidence Interval”.

From the adjustment made with the bivariate and multivariate models, we observed statistically significant coefficients for all estimates made from the inflated zero variables "Surviving larvae".

From the adjustment made of the counting data (Count Part) no significant difference was observed. The coefficients, confidence intervals and standard errors were very low indicating non-significant predictors. It was only in the multivariate analysis that the predictors observed in *Cx*. *pipiens* s.l. and *Anopheles spp*. bioassays presented higher coefficients, confidence intervals and standard errors, but were not significant (p> 0.05).

These observations pointed out the existence of overdispersion of the results due, mainly, to the excess of zeros obtained in bioassays performed with the Bti larvicide.

These observations express therefore a minimal effect of the replica and bioassay variables on the larval survival results obtained in this study.

## Discussion

In this study we evaluated the susceptibility profile of the main culicids of Cabo Verde within the City of Praia, capital of Cabo Verde to *Bacillus thuringiensis* var *israelensis* (Bti) and Temephos in its commercial forms. The results observed indicate a loss of susceptibility, with a difference according to the mosquito species analysed, for Temephos in comparison to Bti with a total susceptibility to the alternative biological compound.

To analyse the robustness of the results obtained in the bioassays performed in this study, the regression models for ZIP and ZINB count variables were applied. The values obtained for the counting predictors point to the existence of overdispersion of the results due, mainly, to the excess of zeros obtained in bioassays performed with the Bti larvicide. The values obtained for the zero inflation predictors point out a minimal effect of the replica and bioassay variables on the larval survival results obtained in this study.

In our experiments, we collected 4633 eggs of *Ae*. *aegypti*, a sufficient number for the study which required 1920 larvae in stage L3/L4, and 48 rafts of *Cx*. *pipiens* s.l. which, due to their low hatching rate, required additional larval collections to complete the amount required for the study (1920 L3/L4 larvae). For *Anopheles* spp. we collected 5000 larvae, of which 3960 L3/L4 stage were selected for the study.

### The species-specific differences to Temephos

The species-specific differences observed in the susceptibility to Temephos, can be linked to the bioecology of each mosquito vector and the previous control activities carried out by the Vector Control Programs of Cabo Verde.

*An*. *arabiensis*, of the *An*. *gambiae* complex, has been targeted as the malaria vector in Cabo Verde for centuries [40, 41]. For its control, the larvicide Temephos [25, 26] was introduced. The continuous and non-rotational use of this larvicide could have exerted selective pressure on its populations, that explain the observed resistance of *An*. *arabiensis*, together with the other sympatric anopheline species *Anopheles pretoriensis* to Temephos [26]

The mortality rates observed for both species was 43.1% at the dose recommended by WHO and 69% at the dose applied by the health agents in Cabo Verde.

*Ae*. *aegypti*, the main vector responsible for the transmission of dengue and zika in Cabo Verde [42, 43], was the targeted species by the vector control programs since the first dengue outbreak in 2009/10 [6]. Thereafter, intervention measures against vectors have increased both in the quantity of used insecticide and the extent of the areas treated with Temephos by health agents, with emphasis on the City of Praia, the focus of outbreaks and epidemics of vector-borne diseases [44, 45].

*Cx*. *pipiens* s.l. is not considered as a vector for mosquito-borne diseases like in Cabo Verde. However, it is a potential vector for diseases such as West Nile, Rift Valley fever and lymphatic filariasis [46–49]. For the latter the identified vector is *An*. *arabiensis* [3]. Although its control is not important for the health authorities, its populations have been submitted to Temephos pressure in the city of Praia where it breeds sympathetically with *Ae*. *aegypti* and *Anopheles* spp. in peri-domestic areas and in non-drinking water. *Cx*. *pipiens* s.l., until the expansion of *Ae*. *aegypti* in recent years [50], was the most abundant mosquito in the city of Praia [41], which explains the abundance of this species in breeding sites that are normally occupied by other species.

For *Ae*. *aegypti* an opposite situation happens to the one described for *Cx*. *pipiens* s.l. in relation to the existence of selective pressure on the survival of the larvae due to the use of Temephos. The main breeding sites for *Ae*. *aegypti* are either small or medium size domestic containers filled with drinking water, which normally are not treated with the larvicide or if they are, the residual effect decreases substantially due to the constant change of water in the majority of the households. This effect was observed in a study performed for *Ae*. *aegypti* in Argentina [51]. This situation, as well as the introduction of its control later than that of *Anopheles*, may explain the greater susceptibility to Temephos observed for this species in relation to *Cx*. *pipiens* s.l. and *Anopheles* spp. In this study *Ae*. *aegypti* presents a mean mortality rate of 90.9% and 98.3% at the dose recommended by the WHO and the dose applied by the health agents, respectively. This reduced susceptibility should be subjected to investigation and can easily evolve for low resistance to Temephos in this vector if the use of this larvicide is maintained. In fact, results of [28] demonstrated low resistance of the *Ae*. *aegypti* populations from the City of Praia to Temephos, in 2012 and 2015. The loss of this low resistance, three years after, could be the result of the decreased use of the larvicide and the fitness cost that arise by maintaining the metabolic mechanism of resistance to Temephos in *Ae*. *aegypti* populations [52, 53]. In Cuba, [54] proved the reversal of the resistance to Temephos in an *Ae*. *aegypti* laboratory strain after six generations without insecticide selection.

Resistance of *Ae*. *aegypti* populations to Temephos is reported from many parts of the world: Brazil from all its territory [55–58], Paraguay [59], the Caribbean from Tortola, Guadalupe and Saint Martin islands [60, 61], in Asia from Thailand, India, Saudi Arabia and Pakistan [62–65]. However, from continental Africa there are no record of resistance to Temephos except for the archipelago of Cabo Verde [28] and the French overseas department of Mayotte [66]. The low detection of resistance to Temephos in Africa, in addition to the lack of further studies in this area, may be explained by the fact that most of the vector-borne disease programs on this continent are focused on combating, mainly, the disease and the vectors of malaria [67–69]. The malaria vector control programs that target mainly Anopheline species have no significant effect on arbovirus vectors [70].

For the *Cx*. *pipiens* s.l., the observed lethality of Temephos in this study was 79%, at the dose recommended by the WHO and 92.9% at the application dose used by local health agents. These results indicate resistance to Temephos of this species, at the standard dose recommended by the WHO and reduced susceptibility, at the dose of application by the Cabo Verde health agents. Thus, Temephos can no longer be considered as an effective insecticide to control *Cx*. *pipiens* s.l. in the City of Praia, and there is a need to confirm the presence of resistant genes in the vector. The loss of susceptibility to this organophosphate as well as the molecular and metabolic mechanisms that lead to this has been studied for a long time, by identifying populations resistant to this product in different parts of the world, such as: Italy [71], Corsica [72], French Polynesia [73], Martinique [74, 75], Portugal [76], China [77], Japan [78], Cyprus [79], Greece [80] and Iran [81], as well as on the African continent: Tunisia [82, 83], Ivory Coast, Burkina Faso [84], Egypt [85], Mayotte island [67] and Morocco[86].

The results discussed so far suggest that the different Temephos resistance profiles, observed among the different culicids analysed, are more related to the pressure of use of the insecticide than to the type of mosquito. This is confirmed in the bioassays performed for populations of *Anopheles* from different locations in the city of Praia, Várzea and Achada Grande Trás. In these sites, it was observed that the difference in susceptibility to Temephos was not due to the existence of different species of anophelines at the sites studied (Fig 9). Other factors not identified in this study, such as the existence of a selective pressure of the insecticide, could explain this result.

Although it is not registered in scientific publications, it is known that the Várzea breeding sites, from which larvae from anopheles bioassays were collected, are subjected to continuous and intense use of Temephos, carried out by health agents in these locations [87–89].

Regardless of the difference in susceptibility to the Temephos species-specific observed in our study, in all bioassays an inverse correlation was confirmed between insecticide susceptibility and the concentration of product applied.

In this study, the concentration currently measured by health agents in vector control was considered as discriminant concentration of Temephos (1 mg / L) and was compared with the diagnostic dose recommended by WHO for the control of *Anopheles* (0.25 mg / L). Temephos discriminating doses for susceptibility monitoring of each potential vector were not defined because the bioassays were performed with the commercial product and not with the technical grade insecticide Temephos. The commercial Temephos product was selected for the bioassays because the objective of the study was to know the current susceptibility of the potential mosquito vectors of Cabo Verde to the larvicidal product applied in control activities.

### Effect of *Bacillus thuringiensis* var *israelensis* (Bti)

For Bti biolarvicide, we observed that the populations of the main Culicidae species in the city of Praia are susceptible to all concentrations analysed, with mean mortality rates of 100% for *Cx*. *pipiens* s.l. and *Anopheles* spp., after 24 h of exposure, and 99.6% for *Ae*. *aegypti*. In Burkina Faso and Benin, treatment with Bti, with the same commercial product Vectobac GR as in Cabo Verde, was effective on larvae of *An*. *gambiae* complex and *Cx*. *quinquefasciatus* [90, 91], and during a study conducted in China where the toxicity of Bti was demonstrated for larvae of *Aedes*, *Culex* and *Anopheles*, especially for the last two [92]. In Kenya and in India, [93, 94] also observed susceptibility to Bti on the larvae of the last stages of *An*. *gambiae* complex and *Cx*. *quinquefasciatus*, more effectively for the anopheline species and with an effective dose dependence on the type of water whether clean or residual. In Uzbekistan, Malaysia and Australia, the larvae of the *Ae*. *aegypti* and *Cx*. *quinquefasciatus* were susceptible to Bti, both in their granulated and liquid formulations [95–97]. The effectiveness of Bti in controlling the vector of malaria has been analysed and demonstrated in different places inside and outside the African continent like in Burkina Faso [98], Ghana [99], Gambia [100], Côte d’Ivoire [101] and Eritrea [102] and in the American continent in Peru and Ecuador [103]. For *Ae*. *aegypti*, the efficacy of Bti was observed in Cabo Verde [27], Cambodia [104, 105], Cuba [106], Florida [107] and Brazil. In the latter, the absence of resistance of different populations to the product [108] was determined with greater effectiveness in its granulated form [109]. The use of Bti showed a great success to control *Cx*. *pipiens* s.l. in different places like in India [110], Florida [111], Turkey [112], Poland and Germany [113].

In this study, we observed the susceptibility of Bti to the main culicid species of Cabo Verde. However, new bioassays are needed to define the discriminating concentrations of Bti and Temephos for each species in order to compare the efficacy of these two larvicides.

### Effect of Temephos

The results obtained on the susceptibility of *Anopheles spp*. to Temephos, based on the bioassays carried out using the WHO methodology [37], were similar to those obtained with the methodology adopted in this study. A mean mortality rate of 60.4% of *Anopheles* spp. (minimum 51.3% and maximum 73.8%) according to the experimental approach of this study was observed. Considering the results obtained from the use of the two methods, the populations of anopheline mosquitoes in the city of Praia are resistant to Temephos, demonstrating the repeatability of the results and validating the method selected in this study. It is important to also note that the method adopted for this study allowed the analysis of larger samples of mosquitoes without affecting the validity of the results. It is thus considered a valid method in assessing the susceptibility of larvae of mosquito populations in Cabo Verde to insecticides.

To determine if the resistance of *Anopheles spp*. to Temephos observed in this study was entirely due to the larvicide and not to other factors that could interfere with the bioassay, the potential influence of the presence of food, the type of water used and the locality of larval origin was analysed. Our results showed that the last two factors could affect the results, with increasing larval mortality in bioassays performed with water from the breeding site and especially those from the locality of Várzea. The presence or absence of food in bioassays with *Anopheles spp*. showed no significant effect on larval mortality, with 51% and 56% mortality respectively observed after 24 hours (Table 2). In a study conducted by [114] on the factors that affect the resistance to DDT on *Anopheles* populations, it is shown that the age of mosquitoes is an important factor, but that larval feeding only exerts a statistically significant low effect on those populations of mosquitoes that are already resistant. Another study conducted on *Cx*. *quinquefasciatus*, susceptibility to Temephos for larvae in stage L2 is influenced by the type of diet (protein or carbohydrate), but this effect was not observed for L3/L4 larvae [115]. Bioassay tests on laboratory strains performed on *Anopheles spp*. using different types of diet (fish food and cat food) were carried out but no difference was observed (unpublished data). The type of water used in the susceptibility bioassay of *Anopheles spp*. to Temephos produced differences in larval mortality. In the bioassays performed with water from the public supply system and with mineral water, mortality rates of 57% and 65.7% were observed, respectively, maintaining the repeatability of the results already observed in the bioassays performed with Temephos (Table 2). In those carried out with water from the breeding sites where the larvae came from, the mortality rate was higher (80.2%). The differences observed could be related to the differences observed in the physiochemical characterization of the water of the breeding sites. This concern mainly salinity, total dissolved solids (TDS) and conductivity. A study by [116] showed a positive relationship between the toxicity of Temephos and the degree of salinity of the water. On the influence of water conductivity on the survival of *An*. *gambiae* complex larvae, [117] observed that the increase in this parameter affects negatively their survival. In addition to the physiochemical parameters analysed in this work, it is necessary to evaluate other biotic and abiotic factors such as the presence of predators, vegetation, water turbidity and ion concentrations to make a more complete identification of the factors that affect the survival of anopheline larvae in Cabo Verde.

The location of breeding sites also influences the mortality of *Anopheles* spp. by Temephos, as noted above. Indeed, we observed differences between the larvae from Várzea and Achada Grande Trás, with the respective mortality rates of 34.2% and 59%. To determine if the difference observed depend on the type of anopheline species *(An*. *arabiensis* and *An*. *pretoriensis)*, all the samples collected and survivors after the Temephos bioassays, including control, were kept in the laboratory until adulthood and were morphological identified. In Várzea, 100% of the adults corresponded to *An*. *arabiensis*, while in Achada Grande Trás, adults not exposed to Temephos corresponded 71% to *An*. *arabiensis* and 29% to *An*. *pretoriensis*. The respective values from resistant larvae were 72% and 28%. These findings showed that the difference found in the mortality of larvae by Temephos is not due to differences in susceptibility of the two species of anophelines present, but to other factors, mainly different use of Temephos in Varzea and Achada Grande Trás. There could be a positive selective pressure for insecticide resistance in Varzea due to the fact that breeding sites in this area are subjected to greater amounts of Temephos for longer. In Iran, it was shown an occurrence of Temephos resistance in *An*. *stephensi* after prolonged use in some parts of the country [118]. For *Ae*. *aegypti*, [119, 120] indicated that Temephos resistance is unstable in the absence of selection pressure caused by the persistent presence of this insecticide and that, differences in the transcript profiles among different susceptible strains are heritable and due to a selection process and are not caused by immediate insecticide exposure. In addition to the selective pressure exerted by the continuous use of Temephos and its tolerance by anophelines, another factor to consider is the use of fertilizers and pesticides in urban and peri-urban agriculture [121–123], as well as the change of behaviour of the species that are adapted to live in polluted waters that proliferate in urban agglomerations [124]. Studies carried out by [125] showed that *An*. *arabiensis* tolerance to urban contaminated larval habitats was accompanied by resistance to Temephos larvicide.

## Conclusion

This study shows the tolerance to the larvicide Temephos applied in Cabo Verde in different grades, on the main malaria vector *An*. *arabiensis* in the country implying that a great attention should be accorded to its use in the vector control program in the country. On the other hand, it confirms the total susceptibility of these mosquitoes to *Bacillus thuringiensis* var *israelensis*, including its minimum dose, which points to its use as an alternative to vector control. Further, studies characterizing the molecular mechanisms involved in the Temephos tolerance observed, as well as simulated field tests to analyse larval survival over time and validate the susceptibility bioassays are recommended.

## Supporting information

S1 Data (XLSX)

S2 Data (XLSX)

S1 File (DOCX)
