# Supplementary material for: Susceptibility of mosquito vectors of the city of Praia, Cabo Verde, to Temephos and Bacillus thuringiensis var israelensis
Source: PLoS One. 2020 Jun 10;15(6):e0234242. doi: 10.1371/journal.pone.0234242 (PMC7286513; doi:10.1371/journal.pone.0234242)
Supplement: S1 File — (DOCX) [file pone.0234242.s003.docx]

**BIVARIATE MODEL**

**AEDES BIOASSAYS (POISSON ZERO INFLATED)**

. zip aedessurvivinglarvae Replica, inflate(aedessurvivinglarvae) exposure(Insecticide) vce(robust)

Fitting constant-only model:

Iteration 0: log pseudolikelihood = -176.81782

Iteration 1: log pseudolikelihood = -139.20156

Iteration 2: log pseudolikelihood = -134.17028

Iteration 3: log pseudolikelihood = -129.16821

Iteration 4: log pseudolikelihood = -123.06165

Iteration 5: log pseudolikelihood = -121.44209

Iteration 6: log pseudolikelihood = -121.09759

Iteration 7: log pseudolikelihood = -121.01516

Iteration 8: log pseudolikelihood = -120.99922

Iteration 9: log pseudolikelihood = -120.99658

Iteration 10: log pseudolikelihood = -120.99595

Iteration 11: log pseudolikelihood = -120.99581

Iteration 12: log pseudolikelihood = -120.99578

Iteration 13: log pseudolikelihood = -120.99578

Fitting full model:

Iteration 0: log pseudolikelihood = -120.99578

Iteration 1: log pseudolikelihood = -111.46671

Iteration 2: log pseudolikelihood = -111.36017

Iteration 3: log pseudolikelihood = -111.36016

Iteration 4: log pseudolikelihood = -111.36016

Zero-inflated Poisson regression Number of obs = 48

Nonzero obs = 20

Zero obs = 28

Inflation model = logit Wald chi2(1) = 3.64

Log pseudolikelihood = -111.3602 Prob > chi2 = 0.0566

--------------------------------------------------------------------------------------

Robust

aedessurvivinglarvae | Coef. Std. Err. z P>|z| [95% Conf. Interval]

---------------------+----------------------------------------------------------------

aedessurvivinglarvae |

Replica | .0620081 .0325202 1.91 **0.057** -.0017303 .1257464

_cons | 1.704666 .2674342 6.37 0.000 1.180504 2.228827

ln(Insecticide) | 1 (exposure)

---------------------+----------------------------------------------------------------

inflate |

aedessurvivinglarvae | -58.38042 .5400474 -108.10 0.000 -59.43889 -57.32194

_cons | 29.93424 .1909824 156.74 0.000 29.55992 30.30856

**CULEX BIOASSAYS** **(NEGATIVE BINOMIAL ZERO INFLATED) -** **NEWTON-RHAPSON MAXIMIZATED**

. zinb Culexsurvivinglarvae Replica, inflate(Culexsurvivinglarvae) exposure(Insecticide) zip difficult technique(nr)

Fitting zip model:

Iteration 0: log likelihood = -168.16354

Iteration 1: log likelihood = -152.07076

Iteration 2: log likelihood = -151.72456

Iteration 3: log likelihood = -151.7244

Iteration 4: log likelihood = -151.7244

Fitting constant-only model:

Iteration 0: log likelihood = -127.98698

Iteration 1: log likelihood = -117.23531

Iteration 2: log likelihood = -112.98728

Iteration 3: log likelihood = -106.88465

Iteration 4: log likelihood = -102.39602

Iteration 5: log likelihood = -98.877004

Iteration 6: log likelihood = -96.881337

Iteration 7: log likelihood = -96.555595

Iteration 8: log likelihood = -96.475777

Iteration 9: log likelihood = -96.460762

Iteration 10: log likelihood = -96.458149

Iteration 11: log likelihood = -96.457873

Iteration 12: log likelihood = -96.457817

Iteration 13: log likelihood = -96.457805

Iteration 14: log likelihood = -96.457803

Fitting full model:

Iteration 0: log likelihood = -96.457803

Iteration 1: log likelihood = -95.16048

Iteration 2: log likelihood = -94.704856

Iteration 3: log likelihood = -94.703167

Iteration 4: log likelihood = -94.703167

Zero-inflated negative binomial regression Number of obs = 48

Nonzero obs = 25

Zero obs = 23

Inflation model = logit LR chi2(1) = 3.51

Log likelihood = -94.70317 Prob > chi2 = 0.0610

--------------------------------------------------------------------------------------

Culexsurvivinglarvae | Coef. Std. Err. z P>|z| [95% Conf. Interval]

---------------------+----------------------------------------------------------------

Culexsurvivinglarvae |

Replica | .0904149 .0479393 1.89 0.059 -.0035444 .1843743

_cons | 1.307794 .2827058 4.63 0.000 .753701 1.861888

ln(Insecticide) | 1 (exposure)

---------------------+----------------------------------------------------------------

inflate |

Culexsurvivinglarvae | -43.02219 23205.52 -0.00 0.999 -45525.01 45438.96

_cons | 22.5065 16359.92 0.00 0.999 -32042.35 32087.36

---------------------+----------------------------------------------------------------

/lnalpha | -.6403438 .3227742 -1.98 0.047 -1.27297 -.0077179

---------------------+----------------------------------------------------------------

alpha | .5271112 .1701379 .2799989 .9923118

--------------------------------------------------------------------------------------

Likelihood-ratio test of alpha=0: chibar2(01) = 114.04 Pr>=chibar2 = 0.0000

**ANOPHELES BIOASSAYS (NEGATIVE BINOMIAL ZERO INFLATED) -** **NEWTON-RHAPSON MAXIMIZATED**

. zinb Anophelessurvivinglarvae Replica, inflate(Anophelessurvivinglarvae) exposure(Insecticide) zip difficult technique(nr)

Fitting zip model:

Iteration 0: log likelihood = -91.291264

Iteration 1: log likelihood = -89.474112

Iteration 2: log likelihood = -89.470886

Iteration 3: log likelihood = -89.470886

Fitting constant-only model:

Iteration 0: log likelihood = -118.15194 (not concave)

Iteration 1: log likelihood = -91.118641

Iteration 2: log likelihood = -87.479278

Iteration 3: log likelihood = -84.904496

Iteration 4: log likelihood = -83.555602

Iteration 5: log likelihood = -82.40175

Iteration 6: log likelihood = -80.255974

Iteration 7: log likelihood = -80.049097

Iteration 8: log likelihood = -80.010245

Iteration 9: log likelihood = -80.00116

Iteration 10: log likelihood = -79.999195

Iteration 11: log likelihood = -79.998707

Iteration 12: log likelihood = -79.998609

Iteration 13: log likelihood = -79.998588

Iteration 14: log likelihood = -79.998583

Fitting full model:

Iteration 0: log likelihood = -79.998583

Iteration 1: log likelihood = -79.780599

Iteration 2: log likelihood = -79.776129

Iteration 3: log likelihood = -79.776128

Zero-inflated negative binomial regression Number of obs = 45

Nonzero obs = 21

Zero obs = 24

Inflation model = logit LR chi2(1) = 0.44

Log likelihood = -79.77613 Prob > chi2 = 0.5048

------------------------------------------------------------------------------------------

Anophelessurvivinglarvae | Coef. Std. Err. z P>|z| [95% Conf. Interval]

-------------------------+----------------------------------------------------------------

Anophelessurvivinglarvae |

Replica | .0173051 .0256828 0.67 0.500 -.0330323 .0676424

_cons | 2.011075 .1635528 12.30 0.000 1.690518 2.331633

ln(Insecticide) | 1 (exposure)

-------------------------+----------------------------------------------------------------

inflate |

Anophelessurvivinglarvae | -39.12275 13102.15 -0.00 0.998 -25718.86 25640.62

_cons | 20.34374 5345.286 0.00 0.997 -10456.22 10496.91

-------------------------+----------------------------------------------------------------

/lnalpha | -2.077136 .4835578 -4.30 0.000 -3.024892 -1.129381

-------------------------+----------------------------------------------------------------

alpha | .1252885 .0605842 .0485631 .3232334

------------------------------------------------------------------------------------------

Likelihood-ratio test of alpha=0: chibar2(01) = 19.39 Pr>=chibar2 = 0.0000

**ANOPHELES WHO BIOASSAYS (NEGATIVE BINOMIAL ZERO INFLATED) -** **NEWTON-RHAPSON MAXIMIZATED**

. zinb anophelessurvivinglarvae Replica, inflate(anophelessurvivinglarvae) exposure(Insecticide) zip difficult technique(nr)

Fitting zip model:

Iteration 0: log likelihood = -72.469581

Iteration 1: log likelihood = -69.597699

Iteration 2: log likelihood = -69.585281

Iteration 3: log likelihood = -69.585281

Fitting constant-only model:

Iteration 0: log likelihood = -99.359032

Iteration 1: log likelihood = -80.298976

Iteration 2: log likelihood = -76.528396 (not concave)

Iteration 3: log likelihood = -74.129297

Iteration 4: log likelihood = -73.288854

Iteration 5: log likelihood = -72.390182

Iteration 6: log likelihood = -71.913889

Iteration 7: log likelihood = -71.401684

Iteration 8: log likelihood = -71.237055

Iteration 9: log likelihood = -71.201168

Iteration 10: log likelihood = -71.193963

Iteration 11: log likelihood = -71.192408

Iteration 12: log likelihood = -71.192065

Iteration 13: log likelihood = -71.19198

Iteration 14: log likelihood = -71.191963

Iteration 15: log likelihood = -71.19196

Fitting full model:

Iteration 0: log likelihood = -71.19196

Iteration 1: log likelihood = -69.328683

Iteration 2: log likelihood = -69.317933

Iteration 3: log likelihood = -69.31115

Iteration 4: log likelihood = -69.311135

Iteration 5: log likelihood = -69.311135

Zero-inflated negative binomial regression Number of obs = 24

Nonzero obs = 23

Zero obs = 1

Inflation model = logit LR chi2(1) = 3.76

Log likelihood = -69.31113 Prob > chi2 = 0.0524

------------------------------------------------------------------------------------------

anophelessurvivinglarvae | Coef. Std. Err. z P>|z| [95% Conf. Interval]

-------------------------+----------------------------------------------------------------

anophelessurvivinglarvae |

Replica | -.0408073 .0199474 -2.05 0.041 -.0799035 -.001711

_cons | 2.505055 .1428615 17.53 0.000 2.225052 2.785059

ln(Insecticide) | 1 (exposure)

-------------------------+----------------------------------------------------------------

inflate |

anophelessurvivinglarvae | -40.12277 32222.15 -0.00 0.999 -63194.38 63114.14

_cons | 20.17341 24022.04 0.00 0.999 -47062.16 47102.51

-------------------------+----------------------------------------------------------------

/lnalpha | -3.50679 1.56427 -2.24 0.025 -6.572703 -.4408767

-------------------------+----------------------------------------------------------------

alpha | .029993 .0469172 .001398 .643472

------------------------------------------------------------------------------------------

Likelihood-ratio test of alpha=0: chibar2(01) = 0.55 Pr>=chibar2 = 0.2295

**MULTIVARIATE MODEL**

**AEDES BIOASSAY (POISSON ZERO INFLATED)**

zip aedessurvivinglarvae Replica Bioassay Replica, inflate(aedessurvivinglarvae) exposure(Insecticide) vce(robust) iterate(1599> 9)

note: Replica omitted because of collinearity

Fitting constant-only model:

Iteration 0: log pseudolikelihood = -481.08077

Iteration 1: log pseudolikelihood = -200.02673

Iteration 2: log pseudolikelihood = -196.2199

Iteration 3: log pseudolikelihood = -190.97592

Iteration 4: log pseudolikelihood = -188.35056

Iteration 5: log pseudolikelihood = -185.94046

Iteration 6: log pseudolikelihood = -185.49239

Iteration 7: log pseudolikelihood = -185.3988

Iteration 8: log pseudolikelihood = -185.38532

Iteration 9: log pseudolikelihood = -185.38369

Iteration 10: log pseudolikelihood = -185.38337

Iteration 11: log pseudolikelihood = -185.38331

Iteration 12: log pseudolikelihood = -185.38329

Fitting full model:

Iteration 0: log pseudolikelihood = -185.38329

Iteration 1: log pseudolikelihood = -181.69358

Iteration 2: log pseudolikelihood = -181.685

Iteration 3: log pseudolikelihood = -181.685

Zero-inflated Poisson regression Number of obs = 48

Nonzero obs = 41

Zero obs = 7

Inflation model = logit Wald chi2(2) = 2.07

Log pseudolikelihood = -181.685 Prob > chi2 = 0.3547

| Robust

aedessurvivinglarvae | Coef. Std. Err. z P>|z| [95% Conf. Interval]

---------------------+----------------------------------------------------------------

aedessurvivinglarvae |

Replica | .0148796 .0241542 0.62 0.538 -.0324617 .0622208

Bioassay | .1080491 .1261111 0.86 0.392 -.1391241 .3552222

Replica | 0 (omitted)

_cons | 1.897198 .2195113 8.64 0.000 1.466964 2.327432

ln(Insecticide) | 1 (exposure)

---------------------+----------------------------------------------------------------

inflate |

aedessurvivinglarvae | -37.72862 .6973242 -54.10 0.000 -39.09535 -36.36189

_cons | 19.2078 .3819642 50.29 0.000 18.45917 19.95644

--------------------------------------------------------------------------------------

**CULEX BIOASSAY (NEGATIVE BINOMIAL ZERO INFLATED) - NEWTON-RHAPSON MAXIMIZATED**

. zinb Culexsurvivinglarvae Replica Bioassay, inflate(Culexsurvivinglarvae) exposure(Insecticide) zip difficult technique(nr)

Fitting zip model:

Iteration 0: log likelihood = -168.16354

Iteration 1: log likelihood = -141.6774

Iteration 2: log likelihood = -140.69388

Iteration 3: log likelihood = -140.69261

Iteration 4: log likelihood = -140.69261

Fitting constant-only model:

Iteration 0: log likelihood = -127.98698

Iteration 1: log likelihood = -117.23531

Iteration 2: log likelihood = -112.98728

Iteration 3: log likelihood = -106.88465

Iteration 4: log likelihood = -102.39602

Iteration 5: log likelihood = -98.877004

Iteration 6: log likelihood = -96.881337

Iteration 7: log likelihood = -96.555595

Iteration 8: log likelihood = -96.475777

Iteration 9: log likelihood = -96.460762

Iteration 10: log likelihood = -96.458149

Iteration 11: log likelihood = -96.457873

Iteration 12: log likelihood = -96.457817

Iteration 13: log likelihood = -96.457805

Iteration 14: log likelihood = -96.457803

Fitting full model:

Iteration 0: log likelihood = -96.457803

Iteration 1: log likelihood = -93.720063

Iteration 2: log likelihood = -93.467911

Iteration 3: log likelihood = -93.467486

Iteration 4: log likelihood = -93.467486

Zero-inflated negative binomial regression Number of obs = 48

Nonzero obs = 25

Zero obs = 23

Inflation model = logit LR chi2(2) = 5.98

Log likelihood = -93.46749 Prob > chi2 = 0.0503

--------------------------------------------------------------------------------------

Culexsurvivinglarvae | Coef. Std. Err. z P>|z| [95% Conf. Interval]

---------------------+----------------------------------------------------------------

Culexsurvivinglarvae |

Replica | .1994931 .0802274 2.49 0.013 .0422502 .356736

Bioassay | -.7603529 .4499393 -1.69 0.091 -1.642218 .1215119

_cons | 1.923783 .4744176 4.06 0.000 .993942 2.853625

ln(Insecticide) | 1 (exposure)

---------------------+----------------------------------------------------------------

inflate |

Culexsurvivinglarvae | -42.81956 22003.37 -0.00 0.998 -43168.64 43083

_cons | 22.42887 15656.46 0.00 0.999 -30663.67 30708.53

---------------------+----------------------------------------------------------------

/lnalpha | -.7584227 .3348661 -2.26 0.024 -1.414748 -.1020973

---------------------+----------------------------------------------------------------

alpha | .4684046 .1568528 .2429868 .9029417

--------------------------------------------------------------------------------------

Likelihood-ratio test of alpha=0: chibar2(01) = 94.45 Pr>=chibar2 = 0.0000

**ANOPHELES BIOASSAY (POISSON ZERO INFLATED)**

. zip Anophelessurvivinglarvae Replica Bioassay, inflate(Anophelessurvivinglarvae) exposure(Insecticide) vce(robust) difficult

Fitting constant-only model:

Iteration 0: log pseudolikelihood = -190.14791

Iteration 1: log pseudolikelihood = -103.49656

Iteration 2: log pseudolikelihood = -99.033047

Iteration 3: log pseudolikelihood = -96.483634

Iteration 4: log pseudolikelihood = -94.969378

Iteration 5: log pseudolikelihood = -93.849111

Iteration 6: log pseudolikelihood = -91.488192

Iteration 7: log pseudolikelihood = -91.336433

Iteration 8: log pseudolikelihood = -91.301243

Iteration 9: log pseudolikelihood = -91.293319

Iteration 10: log pseudolikelihood = -91.29158

Iteration 11: log pseudolikelihood = -91.291303

Iteration 12: log pseudolikelihood = -91.291272

Iteration 13: log pseudolikelihood = -91.291265

Fitting full model:

Iteration 0: log pseudolikelihood = -91.291265

Iteration 1: log pseudolikelihood = -85.023226

Iteration 2: log pseudolikelihood = -84.987081

Iteration 3: log pseudolikelihood = -84.987079

Zero-inflated Poisson regression Number of obs = 45

Nonzero obs = 21

Zero obs = 24

Inflation model = logit Wald chi2(2) = 4.17

Log pseudolikelihood = -84.98708 Prob > chi2 = 0.1240

------------------------------------------------------------------------------------------

| Robust

Anophelessurvivinglarvae | Coef. Std. Err. z P>|z| [95% Conf. Interval]

-------------------------+----------------------------------------------------------------

Anophelessurvivinglarvae |

Replica | .0659824 .0328672 2.01 **0.045** .0015639 .130401

Bioassay | -.3874776 .2390322 -1.62 **0.105** -.8559722 .0810169

_cons | 2.335747 .1980953 11.79 0.000 1.947488 2.724007

ln(Insecticide) | 1 (exposure)

-------------------------+----------------------------------------------------------------

inflate |

Anophelessurvivinglarvae | -40.09426 1.032228 -38.84 0.000 -42.11739 -38.07113

_cons | 19.66363 .2064309 95.26 0.000 19.25903 20.06822

**ANOPHELES WHO BIOASSAY (NEGATIVE BINOMIAL ZERO INFLATED) NEWTON-RHAPSON MAXIMIZATED**

. zinb anophelessurvivinglarvae Replica Bioassay, inflate(anophelessurvivinglarvae) exposure(Insecticide) zip difficult technique> (nr)

Fitting zip model:

Iteration 0: log likelihood = -72.469581

Iteration 1: log likelihood = -68.860447

Iteration 2: log likelihood = -68.840698

Iteration 3: log likelihood = -68.840697

Fitting constant-only model:

Iteration 0: log likelihood = -99.359032

Iteration 1: log likelihood = -80.298976

Iteration 2: log likelihood = -76.528396 (not concave)

Iteration 3: log likelihood = -74.129297

Iteration 4: log likelihood = -73.288854

Iteration 5: log likelihood = -72.390182

Iteration 6: log likelihood = -71.913889

Iteration 7: log likelihood = -71.401684

Iteration 8: log likelihood = -71.237055

Iteration 9: log likelihood = -71.201168

Iteration 10: log likelihood = -71.193963

Iteration 11: log likelihood = -71.192408

Iteration 12: log likelihood = -71.192065

Iteration 13: log likelihood = -71.19198

Iteration 14: log likelihood = -71.191963

Iteration 15: log likelihood = -71.19196

Fitting full model:

Iteration 0: log likelihood = -71.19196

Iteration 1: log likelihood = -69.029698 (not concave)

Iteration 2: log likelihood = -68.724296

Iteration 3: log likelihood = -68.72162

Iteration 4: log likelihood = -68.721619

Zero-inflated negative binomial regression Number of obs = 24

Nonzero obs = 23

Zero obs = 1

Inflation model = logit LR chi2(2) = 4.94

Log likelihood = -68.72162 Prob > chi2 = 0.0846

------------------------------------------------------------------------------------------

anophelessurvivinglarvae | Coef. Std. Err. z P>|z| [95% Conf. Interval]

-------------------------+----------------------------------------------------------------

anophelessurvivinglarvae |

Replica | -.0460504 .0196078 -2.35 0.019 -.0844811 -.0076198

Bioassay | .0653456 .059176 1.10 0.269 -.0506371 .1813284

_cons | 2.375803 .1798684 13.21 0.000 2.023267 2.728338

ln(Insecticide) | 1 (exposure)

inflate |

anophelessurvivinglarvae | -34.12336 7190.899 -0.00 0.996 -14128.03 14059.78

_cons | 17.17395 5361.517 0.00 0.997 -10491.21 10525.55

-------------------------+----------------------------------------------------------------

/lnalpha | -3.907966 2.242069 -1.74 0.081 -8.302341 .4864091

-------------------------+----------------------------------------------------------------

alpha | .0200813 .0450237 .0002479 1.626465

------------------------------------------------------------------------------------------

Likelihood-ratio test of alpha=0: chibar2(01) = 0.24 Pr>=chibar2 = 0.3128
